# Single-cell transcriptomic construction of fibroblast score for analysis of immune infiltration in primary and metastatic ovarian cancer

**DOI:** 10.3389/fgene.2025.1549541

**Published:** 2025-04-28

**Authors:** Qian Guo, Hongmei Ding, Qinlin Zheng

**Affiliations:** Obstetrics and Gynecology Department, The Affiliated Hospital of Southwest Medical University, LuZhou, China

**Keywords:** ovarian cancer, immune infiltration, fibroblast score, single-cell transcriptome, TIMP3

## Abstract

**Purpose:**

Ovarian cancer (OV) is a malignant gynecologic cancer with poor clinical outcomes and poor prognosis. The aim of this study was to explore the immune infiltration between primary and metastatic ovarian cancer and the function of fibroblast differential marker in ovarian cancer immunomodulation.

**Methods:**

Obtained single-cell transcriptome datasets of primary ovarian cancer and metastatic ovarian cancer, performed cell communication analysis and enrichment analysis. Constructed a new fibroblast score, constructed a prognostic model, screened for prognostically relevant fibroblast differential markers, and analyzed the role of differential markers in immune infiltration of ligand-receptor cells.

**Results:**

Single-cell data analysis of ovarian cancer revealed the existence of intercellular communication between fibroblasts and mononuclear macrophages. COX one-way analysis of 28 differential genes in ovarian cancer fibroblasts yielded five genes with prognostic significance for ovarian cancer, and a new Fib score constructed on the basis of these five genes accurately predicted the prognosis of ovarian cancer patients. Further analysis of these five genes revealed that TIMP3 in ovarian cancer fibroblasts affected tumor prognosis, immunosuppression, and drug resistance by targeting M2-type macrophages through the regulation of the CXCL12/CXCR4 signaling axis, which was specifically shown that the higher the expression of TIMP3, the worse the prognosis, the more significant the immune infiltration, and the more drug-resistant the ovarian cancer was.

**Conclusion:**

In metastatic ovarian cancer, fibroblasts induce macrophage polarization through the TIMP3-regulated CXCL signaling pathway, which affects the prognosis of ovarian cancer patients and drug resistance of ovarian cancer cells.

## Introduction

Ovarian cancer (OV) is one of the world’s deadliest malignant tumors of the female reproductive system representing a major and global threat to women’s lives (Siegel et al., 2018), according to Global Cancer Statistics for the year 2020 207,252 patients died of Ovarian Cancer globally in the year 2020 (Sung et al., 2021). OV is one of the most lethal gynecological malignancies. Due to the lack of typical signs and symptoms, most patients are diagnosed at an advanced stage, resulting in a low survival rate (Meyer et al., 2018). Tumor metastasis is closely associated with poor prognosis and is the leading cause of death in patients with ovarian cancer (Jiménez-Sánchez et al., 2017).

Ovarian cancer tumor cell dissemination is the most important mode of ovarian cancer metastasis. A rising number of studies have shown that metastatic tumor cells can produce chemokines or cytokines that reprogram surrounding cells to form the tumor microenvironment and enhance tumor progression, including angiogenesis, inflammation, or epithelial-mesenchymal transition (EMT) (Hübner et al., 2018; Yang and Lin, 2017; Casey et al., 2015). The tumor microenvironment (TME) plays an important role in various cancer types, including OV (Liu et al., 2023), and tumor-associated fibroblasts (CAF), a major component of the tumor stroma, play an important role in the composition and regulation of the tumor microenvironment. A subset of CAF plays a key role in epithelial-mesenchymal transition (EMT) and chemoresistance through direct communication with cancer cells or derived cytokines. chemoresistance (Wang et al., 2018; Huelsken and Hanahan, 2018). In many cancers, CAF is closely associated with the tumor immune microenvironment. CAF alters the immune microenvironment and promotes tumor growth, which makes it a potential therapeutic target (Yu et al., 2022).

It was found that in breast cancer CAF binds IL32 protein derived from CAFs to integrin β3 on the surface of breast cancer cells through cell-to-cell signaling, which activates the downstream p38 MAPK pathway, augments the expression of fibronectin, N-calmodulin, and waviness proteins, and enhances the invasion and metastasis of breast cancer cells (Wen et al., 2019). In hepatocellular carcinoma CAF promotes hepatocellular carcinoma progression by promoting macrophage M2 polarization and inducing PAI-1 secretion via CXCL12 (Chen et al., 2021). In esophageal cancer, FAP-positive MSCs promoted the growth and migration of ESCC cells and peripheral blood monocyte-derived macrophage-like cells, and CAF-like cells induced M2 polarization of macrophage-like cells (Higashino et al., 2019). In ovarian cancer, there is also a close link between CAFs and the tumor microenvironment, e.g., CAFs regulate the ovarian cancer tumor microenvironment to promote tumor progression (Han et al., 2022). CAF-derived CXCL12 could induce the EMT of EOC cells through the CXCR4/Wnt/β-catenin signaling pathway (Zhang et al., 2020). It was found that cisplatin promotes DNA transfer from ovarian cancer cells to CAFs, activates the CGAS-STING-IFNB1 pathway in CAFs, and promotes the release of IFNB1, and STING inhibitors sensitize platinum-based chemotherapy for ovarian cancer by inhibiting the CGAS-STING pathway in CAFs (Liu et al., 2024).IL-33 and culture supernatants derived from CAFs, but not from normal ovarian fibroblasts, lead to higher expression of the M2 macrophage marker genes in human blood monocytes, which promotes macrophage polarization toward M2 and tumor progression (Feng et al., 2022a). Guo et al. (2014) found that CXCL12-CXCR4 Axis promotes proliferation, invasion and metastasis of ovarian cancer, It has also been found that in mice with *in situ* ovarian cancer, the immunosuppressive network was reduced by delivery of a CXCR4 antagonist, which augmented the antitumor immune response and led to tumor-free survival, ultimately generating an antitumor immune response that controlled the growth of metastatic tumors (Komorowski et al., 2016).

Bioinformatics has been widely used in recent years for screening and analysis of genes related to tumorigenesis and development (Xia et al., 2018). During the process of tumor formation, many genes with similar expression patterns or functions actually act in the whole network and influence each other. In our study, single-cell transcriptome data of primary and metastatic ovarian cancers were selected for analysis in the GEO database, and differential genes (THY1, TIMP3, IFI27, CCDC80, DUSP1) of CAF in primary and metastatic ovarian cancers were screened for in-depth analysis, which clarified that the communication between fibroblasts and M2-type macrophages by CXCL12-CXCR4 axis mediated by CAF, which promotes macrophage polarization towards M2. TIMP3 was further identified and validated as a key marker, and TIMP3 promoted macrophage transformation to M2 type. Finally, TIMP3 was found to mediate drug resistance in ovarian cancer in the drug resistance database, and TIMP3 predicted ovarian cancer efficacy to immunotherapy.

## Materials and methods

### Data acquisition and analysis

The gene expression data of this study were obtained from GEO database (Gene Expression Omnibus), we selected the single-cell transcriptome datasets of primary ovarian cancer and metastatic ovarian cancer, GSE130000 and GSE118828, and screened the expression profiles by R software Seurat package, processed them with standardization, homogenization (Harmony), PCA analysis, and analyzed them by ElbowPlot to observe the optimal number of pc, and the positional relationship between each cluster was obtained by TSNE analysis; finally, the cellular clustering and gene expression obtained by setting Find All Markers to identify the marker genes of each cell subtype from the single-cell expression profiles.

### Enrichment analysis

Enrichment analysis of differential genes, including gene ontology (GO) containing biological processes (BP), molecular functions (MF) and cellular components (CC) of different gene functions, and Kyoto Encyclopedia of Genes and Genomes (KEGG) enrichment analysis, to explore the biological functions and signaling pathways of fibroblast-related genes.

### Cell communication

CellChat was able to identify differentially overexpressed ligands and receptors for each cell group, thus predicting important cellular communication, using the R package “CellChat” for cell-to-cell communication analysis and network visualization. Further ligand-receptor and target analysis was performed on two datasets GSE130000 and GSE118828.

### Constructing the prognostic model

First, the condition was set as false discovery rate (FDR) < 0.05, |log2 (Fold Change)| > 1, and the limma package was screened for differentially expressed genes (DEGs) in fibroblasts between primary and metastatic ovarian cancer. Then, the two single-cell data were intersected by taking the differential genes of fibroblasts. In the TCGA database, one-way COX regression analysis was performed to screen prognosis-related genes in ovarian cancer; multifactorial COX analysis was performed to construct a prognosis model. Finally, the differential genes in fibroblasts were scored as high or low, and the patients were divided into high- and low-scoring groups. In the TCGA cohort, the predictive ability of the model was analyzed using Kaplan-Meier curves, and in the survival analysis, the color of the bubbles from blue to red represented the hazard ratio from low to high, and the size of the bubbles was positively correlated with the significance of the Cox P value.

### Relationship between fib score and tumor-associated pathways

The pathway activity scores of 10 tumor-related pathways were calculated for 7,876 cases of 32 tumor types (from the TCGA database) using the R package for GSVA analysis of key genes using the Reverse Phase Protein Array (RPPA) data from the TCPA database (Akbani et al., 2014). These include TSC/mTOR, RTK, RAS/MAPK, PI3K/AKT, hormone ER, hormone AR, EMT, DNA damage response, cell cycle and apoptosis pathways. They are all well-known cancer-related pathways.

### Immune infiltration analysis

Correlation analysis and visualization of infiltrating immune cells were performed using EPIC, MCP and CIBERSORT algorithms. The correlation of TIMP3 with M1 macrophages and M2 macrophages and fibroblasts was analyzed separately.

### Drug sensitivity analysis

Using R software, sensitivity drugs targeting TIMP3 were predicted in the CTRP and PRISM databases. As well as assessing the sensitivity of TIMP3 expression to immunological drugs in different tumor datasets using immunotherapy.

### Statistical analysis

Genes with |log2 fold change| > 1 and FDR <0.05 were defined as Differentially expressed genes in TCGA and GEO data. All statistical analyses were performed using R software (version 4.1.2) and were considered statistically significant at P < 0.05.

## Result

### Cellular communication between fibroblasts and monocytes exists in single-cell data of ovarian cancer

**FIGURE 1:**
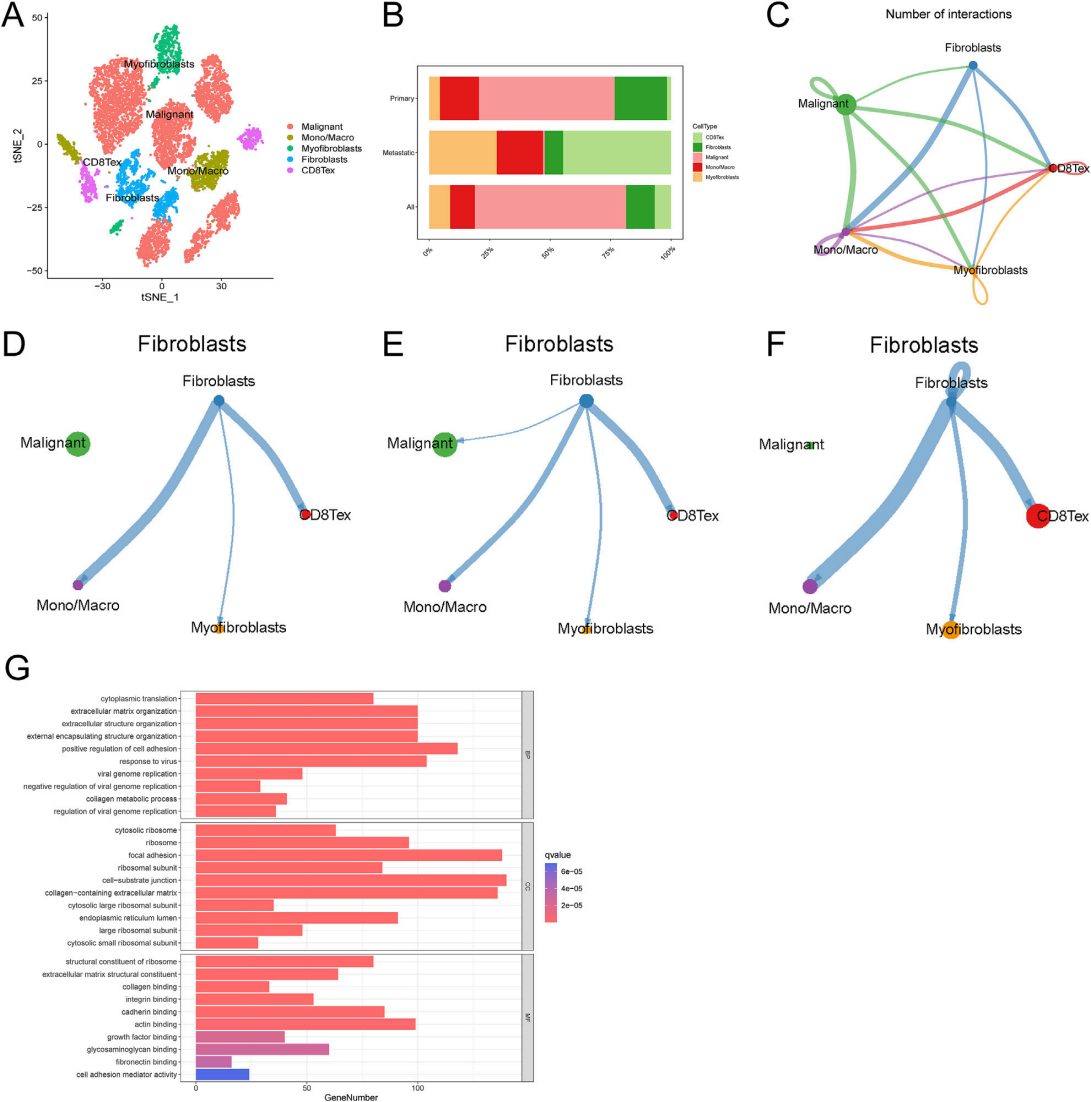
Single-cell data analysis of ovarian cancer. **(A)** Ovarian cancer cell cluster t-SNE **(B)** Cell proportion map in ovarian cancer **(C)** Cell interaction network map **(D–F)** Communication network map of fibroblasts in overall, primary, and metastatic ovarian cancer cells **(G)** Fibroblast enrichment analysis.

We re-annotated the single-cell data GSE130000 (Figure 1A). By analyzing the intercellular communication in this dataset (Figure 1B), we found that fibroblasts had significant communication with monocyte macrophages (Figure 1C). Previous studies have shown a strong relationship between fibroblasts and mononuclear macrophages in metastatic ovarian cancer. We further discussed the relationship between fibroblasts and monocyte macrophages in our dataset and found that intercellular communication existed between fibroblasts and monocyte macrophages in both primary and metastatic as well as primary-only or metastatic-only datasets (Figures 1D–F). Analysis of fibroblasts by enrichment analysis, KEGG analysis of single-cell data showed that fibroblast-associated functions (collagen binding, growth factor binding, fibronectin binding, IL-17 signaling pathway) and CXCL related pathways (PI3K-Akt signaling pathway, IL-17 signaling pathway) were significantly enriched in fibroblasts from metastatic tumors (Figure 1G). Using the same method to study another ovarian cancer single-cell data GSE118828, the results obtained again verified our findings (Supplementary Figures S1A–G). These results suggest the existence of cellular communication between fibroblasts and monocyte macrophages in ovarian cancer patients, in addition to the fact that this communication may be dependent on the CXCL-associated pathway.

### Regulatory molecules that affect cell communication during ovarian cancer cell metastasis

**FIGURE 2:**
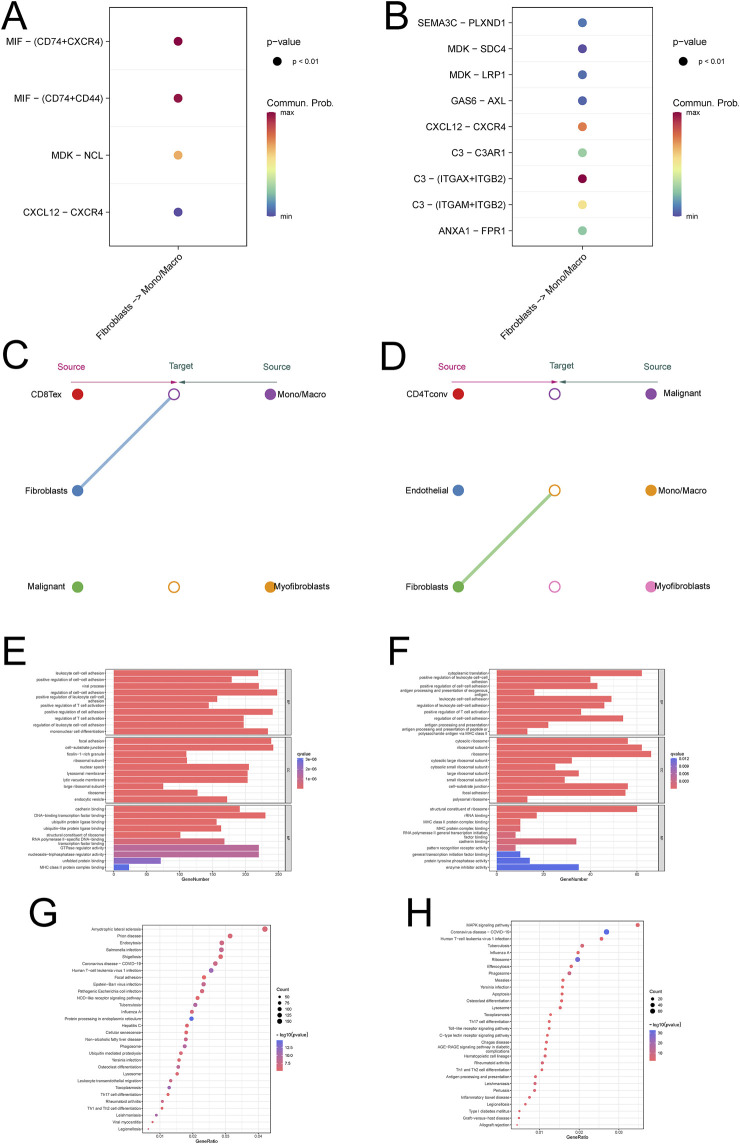
Communication between fibroblasts and macrophages in ovarian cancer **(A, B)** Bubble diagrams are used to demonstrate the correlation of pathways involved in intercellular communication **(C, D)** Hierarchical network diagram of significant cell-cell communication patterns **(E, F)** enrichment analysis of monocyte macrophages **(G, H)** KEGG analysis of monocyte macrophages.

In order to explore the specific molecular regulatory mechanisms of fibroblasts in the process of ovarian cancer metastasis, we performed differential analysis of fibroblast-related differential genes in the two datasets, and the results after taking the intersection showed that 28 differential genes were statistically significant (Figure 2A). Further one-way COX regression analysis yielded 5 genes: THY1, TIMP3, IFI27, CCDC80, DUSP1 (Figure 2B). These five genes had prognostic significance in ovarian cancer (Supplementary Figures S2B–F).

### Construction and validation of a new fib score

We constructed Fib scores h(t) = h0(t) × exp(0.059⋅XTHY1+0.017⋅XTIMP3-0.073⋅XIFI27 + 0.086⋅XCCDC80 + 0.064⋅XDUSP1) for these 5 genes based on Multivariate Cox regression analysis, the higher the Fib score, the worse the prognosis (Figure 2C; Supplementary Figures S2A). We validated the constructed Fib score with the known CAF score and found that the known CAF score was positively correlated with the Fib score and that this CAF score was associated with an immunosuppressive environment (Figure 2D). Enrichment analysis based on fibroblast scores revealed that fibroblast-associated functions (collagen binding, growth factor binding, cadherin binding), CXCL-associated pathways (PI3K-Akt signaling pathway, Ras signaling pathway, Inflammatory mediator regulation of TRP channels), and immune-related pathway (ECM-receptor interaction, ErbB signaling pathway) were significantly enriched in the high Fib score group. Fib score group were significantly enriched, which was consistent with the results of the single-cell data we analyzed (Figures 2E, F). To further explore the relationship between Fib score and tumor microenvironment, we analyzed by multiple algorithms of immune cell infiltration, and the results suggested that fibroblast infiltration was more pronounced in patients with high Fib scores, and more importantly, M2-type macrophages were significantly enriched (Figure 2G). GSVA analysis was performed in pan-cancer (Figure 2H), and the results suggested a significant correlation between high fibroblast score and ovarian cancer prognosis and EMT (Figure 2I). In summary, we constructed and validated a new Fib score that accurately predicted the prognosis of ovarian cancer patients. Meanwhile, we found that progression in metastatic ovarian cancer was achieved by reprogramming of fibroblast-associated genes through regulation of macrophage polarization.

### The CXCL12/CXCR4 signaling axis plays an important role in cellular communication between fibroblasts and monocyte macrophages

**FIGURE 3:**
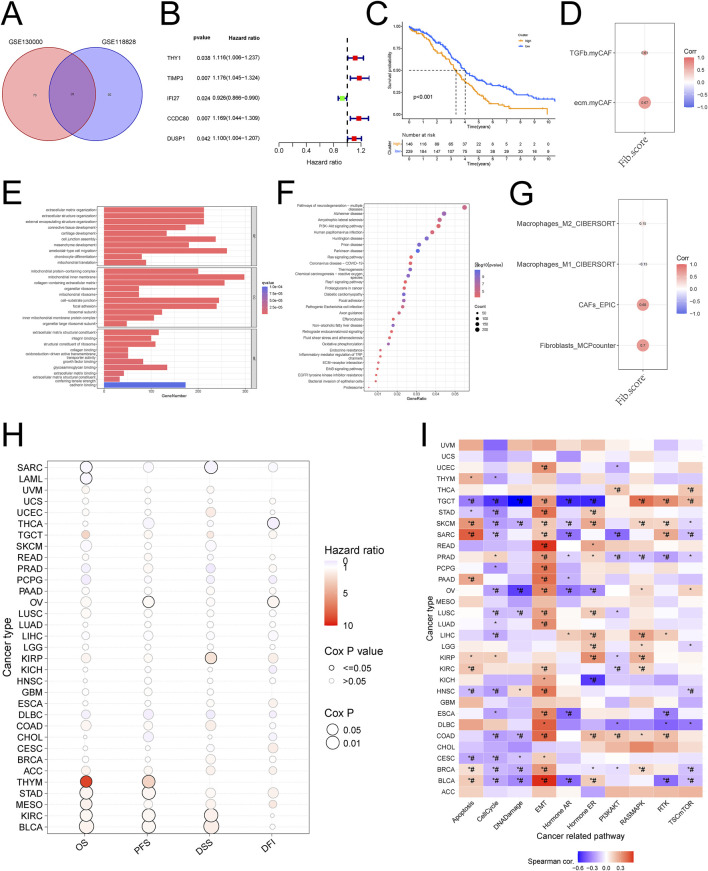
Construction and validation of the new Fib score based on differential genes **(A)** Wayne diagram of differential gene analysis of ovarian cancer fibroblasts **(B)** One-way COX analysis of differential genes **(C)** The new Fib score predicts the prognosis of ovarian cancer **(D)** Correlation analysis of the new Fib score with known immune scores and CAF scores **(E)** Enrichment analysis of the new Fib score **(F)** New KEGG analysis of Fib scores **(G)** Correlation analysis of new Fib scores with tumor immune microenvironment scores **(H)** GSVA scores were used to predict the survival of various tumors **(I)** GSVA scores were used to predict the pathways associated with various tumors.

The initial results of this study showed that there was cellular communication between fibroblasts and monocytes, and in order to further investigate the process of cellular communication, we performed intercellular ligand-receptor pair analysis on single-cell data, which showed that the CXCL signaling pathway was significantly enriched in several single-cell data, and further studies revealed that the CXCL12/CXCR4 signaling axis was most significantly enriched (Figure 3A; Supplementary Figure S3C). Validation revealed that CXCL signaling was mainly emitted by fibroblasts and received by monocyte macrophages as ligands (Figure 3C; Supplementary Figure S3A). Enrichment analysis of monocytes showed that macrophage polarization-associated pathway (NOD-like receptor signaling pathway, Lysosome) was significantly enriched in monocytes from metastatic tumors (Figures 3E, G; Supplementary Figures S3E). More importantly, we also validated this by the single-cell data GSE118828 and found that the relevant pathway (MAPK signaling pathway, Toll-like receptor signaling pathway) was significantly enriched in metastatic ovarian cancer (Figure 3B, D, F, H; Supplementary Figures S3C, D, F). The above results suggest that cellular communication between fibroblasts and monocyte macrophages is dependent on the CXCL12/CXCR4 signaling axis.

### TIMP3 targets CXCL signaling pathway to regulate M2-type macrophages

**FIGURE 4:**
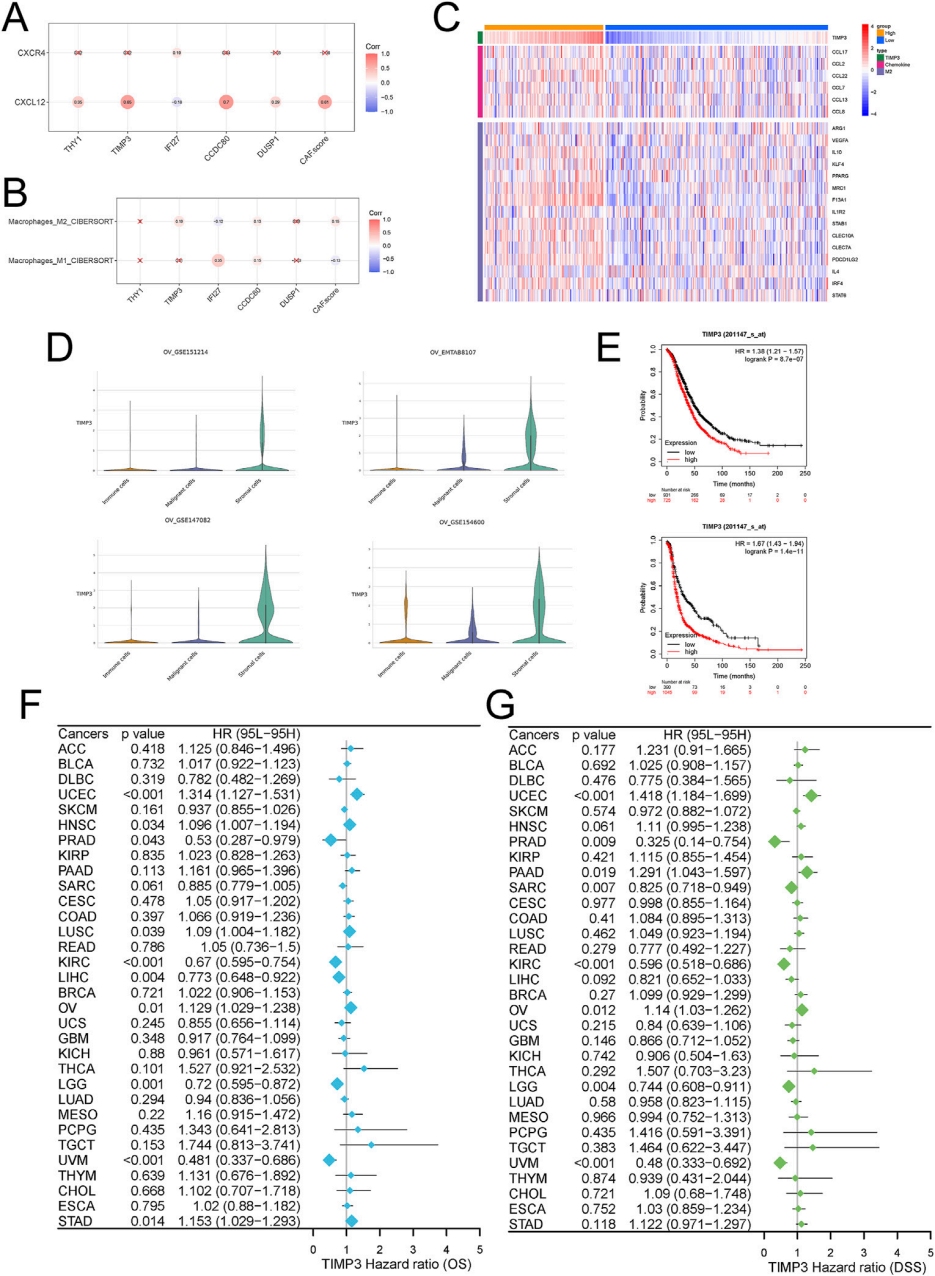
Effects of TIMP3 expression on tumor cells in ovarian cancer **(A)** Correlation analysis of TIMP3 expression with CXCL12/CXCR4 signaling pathway **(B)** Correlation analysis of TIMP3 expression with tumor immune microenvironment **(C)** Heatmap was used to show the correlation between the expression of TIMP3 and chemokine and M2 type macrophage markers **(D)** TIMP3 expression in immune cells immune cells, malignant cells Malignant cells, stromal cells Stromal cells in different ovarian cancer datasets **(E)** Survival analysis of ovarian cancer patients based on TIMP3 expression, Top: OS, Bottom: PFS **(F)** Forest plot used to demonstrate the pan-cancer TIMP3 expression Forest plot used to show the effect of TIMP3 expression in pan-cancer on patients‘ OS. **(G)** Forest plot used to show the effect of TIMP3 expression in pan-cancer on patients’ DSS.

We analyzed the correlation of five genes scored by Fib with targets related to the CXCL signaling pathway and with M1/M2-type macrophages, and showed that TIMP3 was significantly positively correlated with CXCL (Figure 4A). More importantly, TIMP3 was significantly positively correlated with M2-type macrophages (Figures 4B, C). Similarly, TIMP3 was significantly overexpressed in stromal cells in multiple single-cell data (GSE151214, 147082, 154600, EMTAB8107) (Figure 4D).

### TIMP3 expression predicts ovarian cancer prognosis

We further analyzed the effect of TIMP3 expression in ovarian cancer on patient survival. We found that patients with high expression of TIMP3 had a worse prognosis, both in terms of OS and PFS (Figure 4E). The same results were also reflected in survival analysis studies in a variety of tumors (Figures 4F, G). In colorectal cancer (Supplementary Figures S4A, D), head and neck squamous cell carcinoma (Supplementary Figures S4B, E), and breast cancer (Supplementary Figures S4C, F), the predicted survival of patients with high expression of TIMP3 was lower than that predicted by patients with low expression of TIMP3, while at the same time the expression of TIMP3 was negatively correlated with the number of cytotoxic T-lymphocytes, a result which further illustrates that high expressing TIMP3 patients were predicted to have lower survival.

### TIMP3 is strongly associated with CAFs and immune infiltration suppression

**FIGURE 5:**
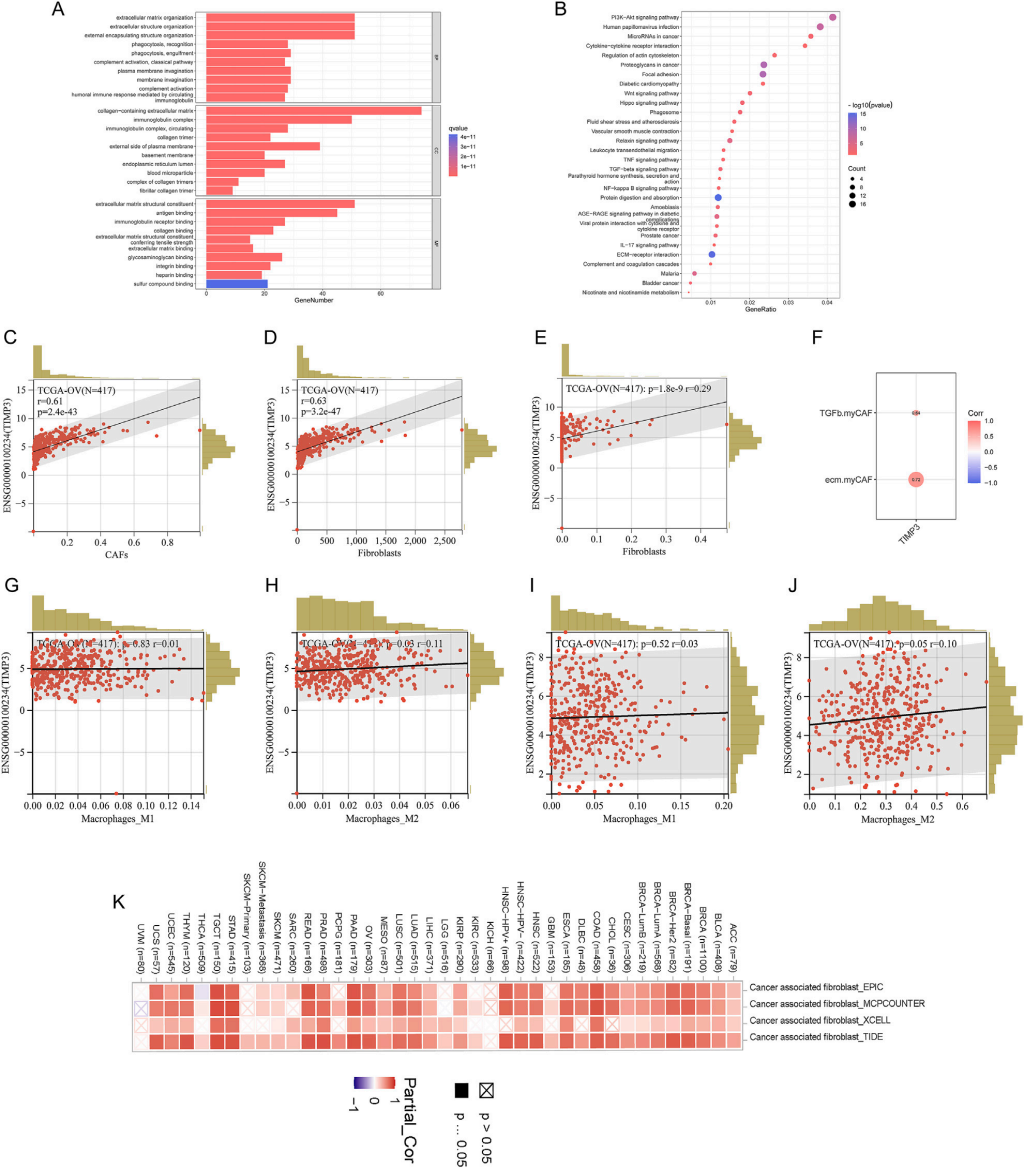
Relationship between TIMP3 and tumor immune microenvironment **(A)** Enrichment analysis of TIMP3 expression in the TCGA ovarian cancer dataset **(B)** KEGG analysis of TIMP3 expression in the TCGA ovarian cancer dataset **(C)** EPIC immune infiltration algorithm to analyze the correlation between TIMP3 expression and immune infiltration of CAFs **(D)** MCP immune infiltration algorithm to Analysis of the correlation between TIMP3 expression and Fib immune infiltration **(E)** Xcell immune infiltration algorithm to analyze the correlation between TIMP3 expression and Fib immune infiltration **(F)** Correlation between TIMP3 expression and known CAF-related immune scores **(G)** CIB immune infiltration algorithm to analyze the correlation between TIMP3 expression and M1-type macrophage immune infiltration **(H)** CIB Immune infiltration algorithm to analyze the correlation between TIMP3 expression and M2-type macrophage immune infiltration **(I)** Xcell immune infiltration algorithm to analyze the correlation between TIMP3 expression and M1-type macrophage immune infiltration **(J)** Xcell immune infiltration algorithm to analyze the correlation between TIMP3 expression and M2-type macrophage immune infiltration **(K)** EPIC, MCP, Xcell, TIDE four immune infiltration algorithms to analyze the correlation between TIMP3 expression and CAFs immune infiltration in pan-cancer.

We grouped high and low TIMP3 expression in the patient dataset for enrichment analysis, and fibroblast-associated known pathways, CXCL-associated pathways, immune-associated pathways, and macrophage polarization (PI3K-Akt signaling pathway, Wnt signaling pathway, TGF -beta signaling pathway, NF-kappa B signaling pathway, IL-17 signaling pathway) were significantly enriched (Figures 5A, B). To further explore the relationship between TIMP3 and the tumor immune microenvironment, the analysis was performed by an algorithm of immune cell infiltration, and we found that the expression of TIMP3 correlated significantly with the immune infiltration of fibroblasts, tumor-associated fibroblasts, and M2-type macrophages, and the higher the expression of TIMP3, the more pronounced the CAFs and M2 infiltration (Figures 5C–E, G, J). More surprisingly, we validated the immune infiltration algorithm by 4 immune infiltration algorithms in pan-cancerous species, and the results also showed that TIMP3 expression correlated with CAFs (Figure 5K). We also validated by known CAF scores and found that there was a significant positive correlation between TIMP3 and CAF scores associated with immunosuppressive environment (Figure 5F). Taken together, we can infer that the expression of TIMP3 correlates with the suppression of tumor immune infiltration, which may be relevant to the immunotherapy of tumors.

### TIMP3 expression in ovarian cancer correlates with tumor drug resistance

**FIGURE 6:**
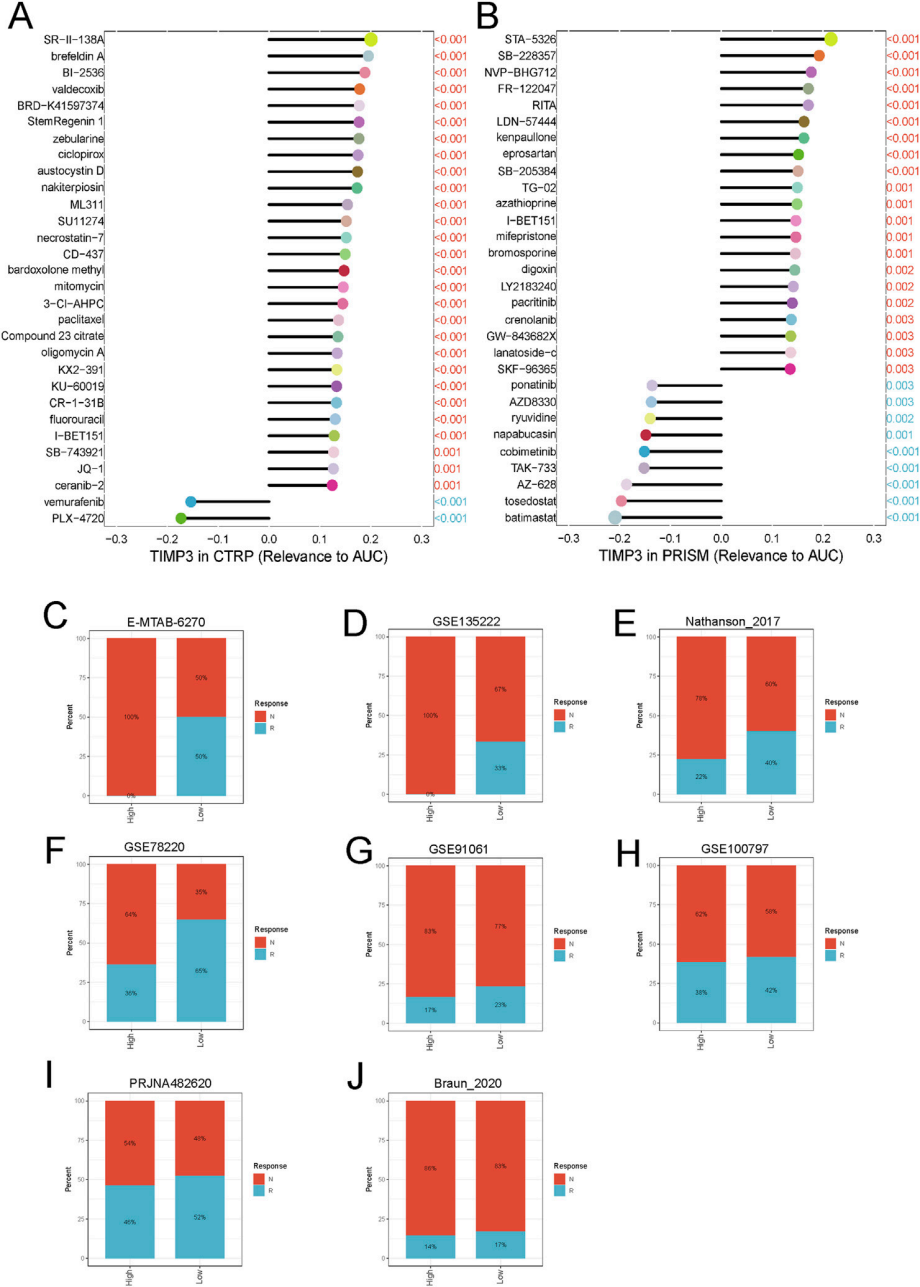
Correlation between TIMP3 and tumor immunosuppression **(A)** TIMP3-based ovarian cancer sensitivity to drugs in the CTRP database **(B)** TIMP3-based ovarian cancer sensitivity to drugs in the PRISM database **(C–J)** Analysis of the relationship between TIMP3 expression and the response to immunotherapy of different tumor cells.

We calculated the correlation of TIMP3 with drug sensitivity in PRISM and CTRP databases to retrieve the relationship of different drugs with fibroblasts and macrophages. The results showed that the expression of TIMP3 was correlated with the resistance of ovarian cancer cells to the drug I-BET151 (Figures 6A, B). Further analysis in different tumor datasets showed that the proportion of tumor cells that were unresponsive to immunotherapeutic drugs was higher in both TIMP3 high expression groups than in the TIPM3 low expression group, and this result demonstrated that high expression of TIMP3 could enhance the immunosuppressive ability of tumor cells, thus enhancing their immunotherapeutic resistance (Figures 6C–J).

## Discussion

Ovarian cancer (OV) is the most lethal gynecologic malignancy worldwide and is on the rise, with the majority of patients being detected when their tumors have already progressed to an advanced stage (Huang et al., 2023). GLOBOCAN 2020 (Sung et al., 2021) shows that in 2020 there were 313,959 new cases and 207,252 new deaths from ovarian cancer worldwide, with a 5-year survival rate of less than the 5-year survival rate is less than 40% and the mortality rate is as high as 60% (Perales-Puchalt et al., 2018). Despite the great progress in surgical and pharmacologic treatments, it is difficult to maintain complete/partial remission in patients with ovarian cancer, and the exploration of reliable biomarkers and precise molecular mechanisms is crucial for the early diagnosis, treatment and prognosis of OV. In recent years, the rapid development of bioinformatics has facilitated the processing of large sample size data, giving researchers new perspectives and ideas in tumor exploration and research.

In this study, we investigated inter-subpopulation communication using two single-cell datasets from two GEO databases containing primary and metastatic ovarian cancers and found significant cellular communication between fibroblasts and monocyte macrophages across datasets as well as across disease states. Fibroblasts were subjected to enrichment analysis revealing that fibroblast-associated known pathways and CXCL-associated pathways were significantly enriched in metastatic ovarian cancer. Differential gene analysis of fibroblasts in primary and metastatic ovarian cancers revealed that THY1, TIMP3, IFI27, CCDC80, and DUSP1 were associated with prognosis, and a prognostic model was constructed. The model was validated using immune infiltration fib score and enrichment analysis was performed, which revealed that fibroblast-related pathways, CXCL-related pathways, and immune-related pathways were enriched. Immune cell infiltration analysis was performed, and the results suggested that fibroblast infiltration was more pronounced in patients with high Fib scores, and more importantly, M2-type macrophages were significantly enriched. Further in-depth analysis of the ligand receptors of fibroblasts and monocyte macrophages revealed that the CXCL12/CXCR4 signaling axis was most significantly enriched. To explore the key genes in the model genes, we studied five genes in depth and found that in metastatic ovarian cancer, fibroblasts induced macrophage polarization to M2-type macrophages through the TIMP3-regulated CXCL signaling pathway, which in turn affected the prognosis of ovarian cancer patients and the drug resistance of ovarian cancer cells.

The tumor microenvironment consists of tumor cells as well as a variety of mesenchymal and immune cells in tumor tissues, which are closely related to tumor growth. The tumor microenvironment is responsible for tumorigenesis and progression, and to some extent influences the efficiency of immunotherapy (Neal et al., 2018). Tumor-associated fibroblasts are the most abundant cell type in the tumor microenvironment, greatly influencing immune cell activity and functioning within the tumor microenvironment (Liu et al., 2023). Numerous studies have shown that increased tumor-associated fibroblasts can be a factor in the poor prognosis of ovarian cancer patients. In the context of fibroblast biology and the tumor microenvironment in ovarian cancer, tumor-associated fibroblasts are a group of non-immune-associated tumor cells that may positively contribute to the ability of tumor cells to proliferate, migrate, and metastasize (Shen et al., 2024). Lin et al. (2022) found that Periostin enhances the M2 through integrin-mediated NF-κB and TGF-β2 signaling pathways. Macrophages and cancer-associated fibroblasts to promote ovarian cancer metastasis. High CD47 expression was found to be closely associated with immune infiltration of ovarian cancer cells, thereby affecting the tumor microenvironment and possibly inducing ovarian cancer heterogeneity (Yu et al., 2021). In our study, we found that the infiltration of tumor-associated fibroblasts was more significant in ovarian cancer tumors that developed metastases compared to those that did not. And fibroblast-related as well as immune-related signaling pathways were significantly enriched in highly scored fibroblasts. In the validation of fibroblasts, the effect of fibroblasts on macrophage polarization toward the M2 type was more pronounced in metastatic ovarian cancers.

Tissue inhibitor of metalloproteinases 3 (TIMP3) is the major endogenous inhibitor of matrix metalloproteinases (MMPs) and inhibits tumor growth, invasion, metastasis and angiogenesis. Previous reports have shown that TIMP3 is associated with the development of a variety of tumors (Huang et al., 2019; Defamie et al., 2015). In previous reports, we observed that TIMP3 showed high expression in pancreatic cancer tissues (Jones et al., 2004), and TIMP3 was upregulated in cervical cancer cell lines and cervical squamous cell carcinoma tissues. Shaker et al. created a model in which they noted that the expression level of TIMP3 was increased in normal cervical cells during carcinogenesis as compared to parental HCK cells (Shaker et al., 2011). We also observed that in renal clear cell carcinoma, by constructing a prognostic model of immune-related genes, screening for six immune-related genes including TIMP3, patients in the high-risk group model had a worse prognosis and the high-risk group was enriched in more immune-related pathways. A negative correlation between the high-risk group and response to immunotherapy was also observed based on the risk score (Zhou et al., 2022). We also observed a positive correlation between TIMP3 and fibroblast-presented expression in the ovarian cancer study (Feng et al., 2022b). In our study, TIMP3 expression was found to be higher in stromal cells and TIMP3 was positively correlated with fibroblasts and M2-type macrophages, and we suggest that TIMP3 overexpression promotes fibroblast-induced macrophage polarization toward the M2 type via the CXCL12/CXCR4 axis. TIMP3 expression was also found to be negatively correlated with immunotherapy response in our dataset.

In conclusion, we analyzed datasets of primary and metastatic ovarian cancer, and fibroblast TIMP3 promotes monocyte macrophage polarization to M2-type through CXCL12/CXCR4 axis signaling in different disease states. Moreover, TIMP3 was associated with ovarian cancer prognosis, positively correlated with tumor resistance, and negatively correlated with immunotherapy response.

Our study also has some limitations, and this study analyzed the dataset based on bioinformatics, which provided new insights into ovarian cancer research. However, this study lacked experiments to prove the bioinformatics results, which will be further improved and deepened in our subsequent studies.

## Data Availability

The original contributions presented in the study are included in the article/Supplementary Material, further inquiries can be directed to the corresponding author.

## References

[B1] Akbani R. Ng P. K. Werner H. M. Shahmoradgoli M. Zhang F. Ju Z. (2014). A pan-cancer proteomic perspective on the Cancer Genome Atlas. Nat. Commun. 5, 3887. 10.1038/ncomms4887 24871328 PMC4109726

[B2] Casey S. C. Amedei A. Aquilano K. Azmi A. S. Benencia F. Bhakta D. (2015). Cancer prevention and therapy through the modulation of the tumor microenvironment. Semin. Cancer Biol. 35 (Suppl. l), S199-S223–S223. 10.1016/j.semcancer.2015.02.007 25865775 PMC4930000

[B3] Chen S. Morine Y. Tokuda K. Yamada S. Saito Y. Nishi M. (2021). Cancer-associated fibroblast-induced M2-polarized macrophages promote hepatocellular carcinoma progression via the plasminogen activator inhibitor-1 pathway. Int. J. Oncol. 59 (2), 59. 10.3892/ijo.2021.5239 34195849 PMC8253588

[B4] Defamie V. Sanchez O. Murthy A. Khokha R. (2015). TIMP3 controls cell fate to confer hepatocellular carcinoma resistance. Oncogene 34 (31), 4098–4108. 10.1038/onc.2014.339 25347747

[B5] Feng C. Kou L. Yin P. Jing Y. (2022a). Excessive activation of IL-33/ST2 in cancer-associated fibroblasts promotes invasion and metastasis in ovarian cancer. Oncol. Lett. 23 (5), 158. 10.3892/ol.2022.13278 35399326 PMC8987947

[B6] Feng S. Xu Y. Dai Z. Yin H. Zhang K. Shen Y. (2022b). Integrative analysis from multicenter studies identifies a WGCNA-derived cancer-associated fibroblast signature for ovarian cancer. Front. Immunol. 13, 951582. 10.3389/fimmu.2022.951582 35874760 PMC9304893

[B7] Guo Q. Gao B. L. Zhang X. J. Liu G. C. Xu F. Fan Q. Y. (2014). CXCL12-CXCR4 Axis promotes proliferation, migration, invasion, and metastasis of ovarian cancer. Oncol. Res. 22 (5-6), 247–258. 10.3727/096504015X14343704124430 26629936 PMC7842602

[B8] Han L. Guo X. Du R. Guo K. Qi P. Bian H. (2022). Identification of key genes and pathways related to cancer-associated fibroblasts in chemoresistance of ovarian cancer cells based on GEO and TCGA databases. J. Ovarian Res. 15 (1), 75. 10.1186/s13048-022-01003-2 35739532 PMC9219195

[B9] Higashino N. Koma Y. I. Hosono M. Takase N. Okamoto M. Kodaira H. (2019). Fibroblast activation protein-positive fibroblasts promote tumor progression through secretion of CCL2 and interleukin-6 in esophageal squamous cell carcinoma. Lab. Invest 99 (6), 777–792. 10.1038/s41374-018-0185-6 30683902

[B10] Huang H. L. Liu Y. M. Sung T. Y. Huang T. C. Cheng Y. W. Liou J. P. (2019). TIMP3 expression associates with prognosis in colorectal cancer and its novel arylsulfonamide inducer, MPT0B390, inhibits tumor growth, metastasis and angiogenesis. Theranostics 9 (22), 6676–6689. 10.7150/thno.34020 31588243 PMC6771239

[B11] Huang W. Wu Y. Luo N. Shuai X. Guo J. Wang C. (2023). Identification of TRPM2 as a prognostic factor correlated with immune infiltration in ovarian cancer. J. Ovarian Res. 16 (1), 169. 10.1186/s13048-023-01225-y 37608401 PMC10463424

[B12] Hübner M. Hinske C. L. Effinger D. Wu T. Thon N. Kreth F. W. (2018). Intronic miR-744 inhibits glioblastoma migration by functionally antagonizing its host gene MAP2K4. Cancers (Basel) 10 (11), 400. 10.3390/cancers10110400 30366472 PMC6266622

[B13] Huelsken J. Hanahan D. (2018). A subset of cancer-associated fibroblasts determines therapy resistance. Cell 172 (4), 643–644. 10.1016/j.cell.2018.01.028 29425485

[B14] Jiménez-Sánchez A. Memon D. Pourpe S. Veeraraghavan H. Li Y. Vargas H. A. (2017). Heterogeneous tumor-immune microenvironments among differentially growing metastases in an ovarian cancer patient. Cell 170 (5), 927–938.e20. 10.1016/j.cell.2017.07.025 28841418 PMC5589211

[B15] Jones L. E. Humphreys M. J. Campbell F. Neoptolemos J. P. Boyd M. T. (2004). Comprehensive analysis of matrix metalloproteinase and tissue inhibitor expression in pancreatic cancer: increased expression of matrix metalloproteinase-7 predicts poor survival. Clin. Cancer Res. 10 (8), 2832–2845. 10.1158/1078-0432.ccr-1157-03 15102692

[B16] Komorowski M. P. McGray A. R. Kolakowska A. Eng K. Gil M. Opyrchal M. (2016). Reprogramming antitumor immunity against chemoresistant ovarian cancer by a CXCR4 antagonist-armed viral oncotherapy. Mol. Ther. Oncolytics 3, 16034. 10.1038/mto.2016.34 28035333 PMC5155641

[B17] Lin S. C. Liao Y. C. Chen P. M. Yang Y. Y. Wang Y. H. Tung S. L. (2022). Periostin promotes ovarian cancer metastasis by enhancing M2 macrophages and cancer-associated fibroblasts via integrin-mediated NF-κB and TGF-β2 signaling. J. Biomed. Sci. 29 (1), 109. 10.1186/s12929-022-00888-x 36550569 PMC9784270

[B18] Liu H. Zhou L. Cheng H. Wang S. Luan W. Cai E. (2023). Characterization of candidate factors associated with the metastasis and progression of high-grade serous ovarian cancer. Chin. Med. J. Engl. 136 (24), 2974–2982. 10.1097/CM9.0000000000002328 37284741 PMC10752471

[B19] Liu J. Liu C. Ma Y. Pan X. Chu R. Yao S. (2024). STING inhibitors sensitize platinum chemotherapy in ovarian cancer by inhibiting the CGAS-STING pathway in cancer-associated fibroblasts (CAFs). Cancer Lett. 588, 216700. 10.1016/j.canlet.2024.216700 38373690

[B20] Meyer L. A. He W. Sun C. C. Zhao H. Wright A. A. Suidan R. S. (2018). Neoadjuvant chemotherapy in elderly women with ovarian cancer: rates of use and effectiveness. Gynecol. Oncol. 150 (3), 451–459. 10.1016/j.ygyno.2018.06.020 29961559 PMC8328050

[B21] Neal J. T. Li X. Zhu J. Giangarra V. Grzeskowiak C. L. Ju J. (2018). Organoid modeling of the tumor immune microenvironment. Cell 175 (7), 1972–1988. 10.1016/j.cell.2018.11.021 30550791 PMC6656687

[B22] Perales-Puchalt A. Perez-Sanz J. Payne K. K. Svoronos N. Allegrezza M. J. Chaurio R. A. (2018). Frontline Science: microbiota reconstitution restores intestinal integrity after cisplatin therapy. J. Leukoc. Biol. 103 (5), 799–805. 10.1002/JLB.5HI1117-446RR 29537705 PMC6004318

[B23] Shaker M. Yokoyama Y. Mori S. Tsujimoto M. Kawaguchi N. Kiyono T. (2011). Aberrant expression of disintegrin-metalloprotease proteins in the formation and progression of uterine cervical cancer. Pathobiology 78 (3), 149–161. 10.1159/000324314 21613802

[B24] Shen L. Li A. Cui J. Liu H. Zhang S. (2024). Integration of single-cell RNA-seq and bulk RNA-seq data to construct and validate a cancer-associated fibroblast-related prognostic signature for patients with ovarian cancer. J. Ovarian Res. 17 (1), 82. 10.1186/s13048-024-01399-z 38627854 PMC11020192

[B25] Siegel R. L. Miller K. D. Jemal A. (2018). Cancer statistics, 2018. CA Cancer J. Clin. 68 (1), 7–30. 10.3322/caac.21442 29313949

[B26] Sung H. Ferlay J. Siegel R. L. Laversanne M. Soerjomataram I. Jemal A. (2021). Global cancer statistics 2020: GLOBOCAN estimates of incidence and mortality worldwide for 36 cancers in 185 countries. CA Cancer J. Clin. 71 (3), 209–249. 10.3322/caac.21660 33538338

[B27] Wang L. Zhang F. Cui J. Y. Chen L. Chen Y. T. Liu B. W. (2018). CAFs enhance paclitaxel resistance by inducing EMT through the IL-6/JAK2/STAT3 pathway. Oncol. Rep. 39 (5), 2081–2090. 10.3892/or.2018.6311 29565447 PMC5928760

[B28] Wen S. Hou Y. Fu L. Xi L. Yang D. Zhao M. (2019). Cancer-associated fibroblast (CAF)-derived IL32 promotes breast cancer cell invasion and metastasis via integrin β3-p38 MAPK signalling. Cancer Lett. 442, 320–332. 10.1016/j.canlet.2018.10.015 30391782

[B29] Xia L. Su X. Shen J. Meng Q. Yan J. Zhang C. (2018). *ANLN* functions as a key candidate gene in cervical cancer as determined by integrated bioinformatic analysis. Cancer Manag. Res. 10, 663–670. 10.2147/CMAR.S162813 29670400 PMC5896649

[B30] Yang L. Lin P. C. (2017). Mechanisms that drive inflammatory tumor microenvironment, tumor heterogeneity, and metastatic progression. Semin. Cancer Biol. 47, 185–195. 10.1016/j.semcancer.2017.08.001 28782608 PMC5698110

[B31] Yu L. Ding Y. Wan T. Deng T. Huang H. Liu J. (2021). Significance of CD47 and its association with tumor immune microenvironment heterogeneity in ovarian cancer. Front. Immunol. 12, 768115. 10.3389/fimmu.2021.768115 34966389 PMC8710451

[B32] Yu L. Shen N. Shi Y. Shi X. Fu X. Li S. (2022). Characterization of cancer-related fibroblasts (CAF) in hepatocellular carcinoma and construction of CAF-based risk signature based on single-cell RNA-seq and bulk RNA-seq data. Front. Immunol. 13, 1009789. 10.3389/fimmu.2022.1009789 36211448 PMC9537943

[B33] Zhang F. Cui J. Y. Gao H. F. Yu H. Gao F. F. Chen J. L. (2020). Cancer-associated fibroblasts induce epithelial-mesenchymal transition and cisplatin resistance in ovarian cancer via CXCL12/CXCR4 axis. Future Oncol. 16 (32), 2619–2633. 10.2217/fon-2020-0095 32804554

[B34] Zhou L. Fang H. Yin M. Long H. Weng G. (2022). Novel immune-related signature based on immune cells for predicting prognosis and immunotherapy response in clear cell renal cell carcinoma. J. Clin. Lab. Anal. 36 (6), e24409. 10.1002/jcla.24409 35441741 PMC9169179

