# Supplementary material for: Single-cell transcriptomic construction of fibroblast score for analysis of immune infiltration in primary and metastatic ovarian cancer
Source: Front Genet. 2025 Apr 28;16:1549541. doi: 10.3389/fgene.2025.1549541 (PMC12066613; doi:10.3389/fgene.2025.1549541)
Supplement: Supplementary file 1 [file Image1.pdf]

FigureS1

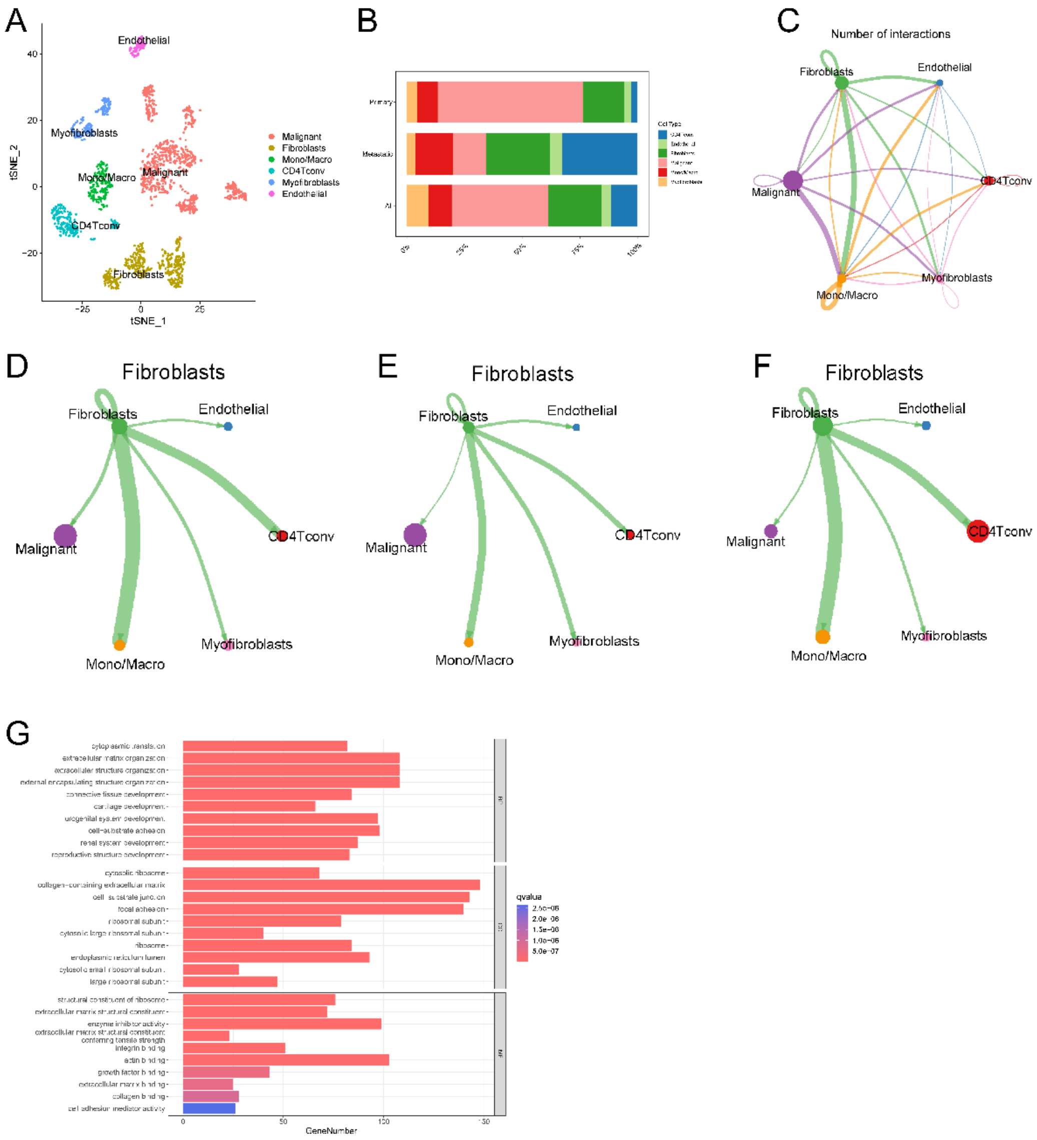
A
B
C
Number of interactions
D
E
F
Fibroblasts
G
tSNE_1
tSNE_2
Malignant
Fibroblasts
Mono/Macro
CD4Tconv
Myofibroblasts
Endothelial
GeneNumber
qvalue

FigureS2

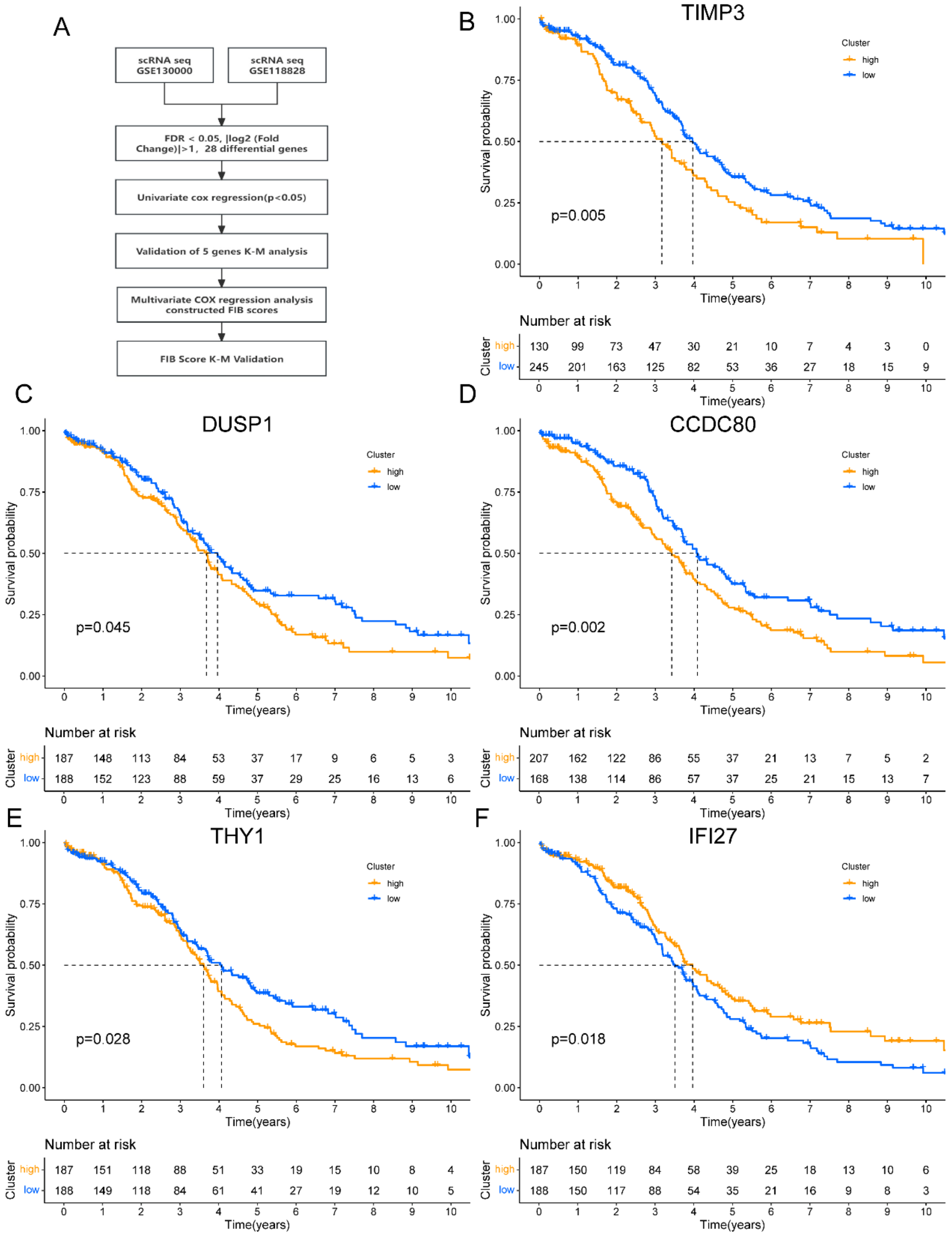
A
scRNA seq GSE130000
scRNA seq GSE118828
FDR < 0.05, |log2 (Fold Change)|>1, 28 differential genes
Univariate cox regression(p<0.05)
Validation of 5 genes K-M analysis
Multivariate COX regression analysis constructed FIB scores
FIB Score K-M Validation
B
TIMP3
p=0.005
Cluster
high
low
Survival probability
Time(years)
Number at risk
high 130 99 73 47 30 21 10 7 4 3 0
low 245 201 163 125 82 53 36 27 18 15 9
C
DUSP1
p=0.045
Number at risk
high 187 148 113 84 53 37 17 9 6 5 3
low 188 152 123 88 59 37 29 25 16 13 6
D
CCDC80
p=0.002
Number at risk
high 207 162 122 86 55 37 21 13 7 5 2
low 168 138 114 86 57 37 25 21 15 13 7
E
THY1
p=0.028
Number at risk
high 187 151 118 88 51 33 19 15 10 8 4
low 188 149 118 84 61 41 27 19 12 10 5
F
IFI27
p=0.018
Number at risk
high 187 150 119 84 58 39 25 18 13 10 6
low 188 150 117 88 54 35 21 16 9 8 3

FigureS3

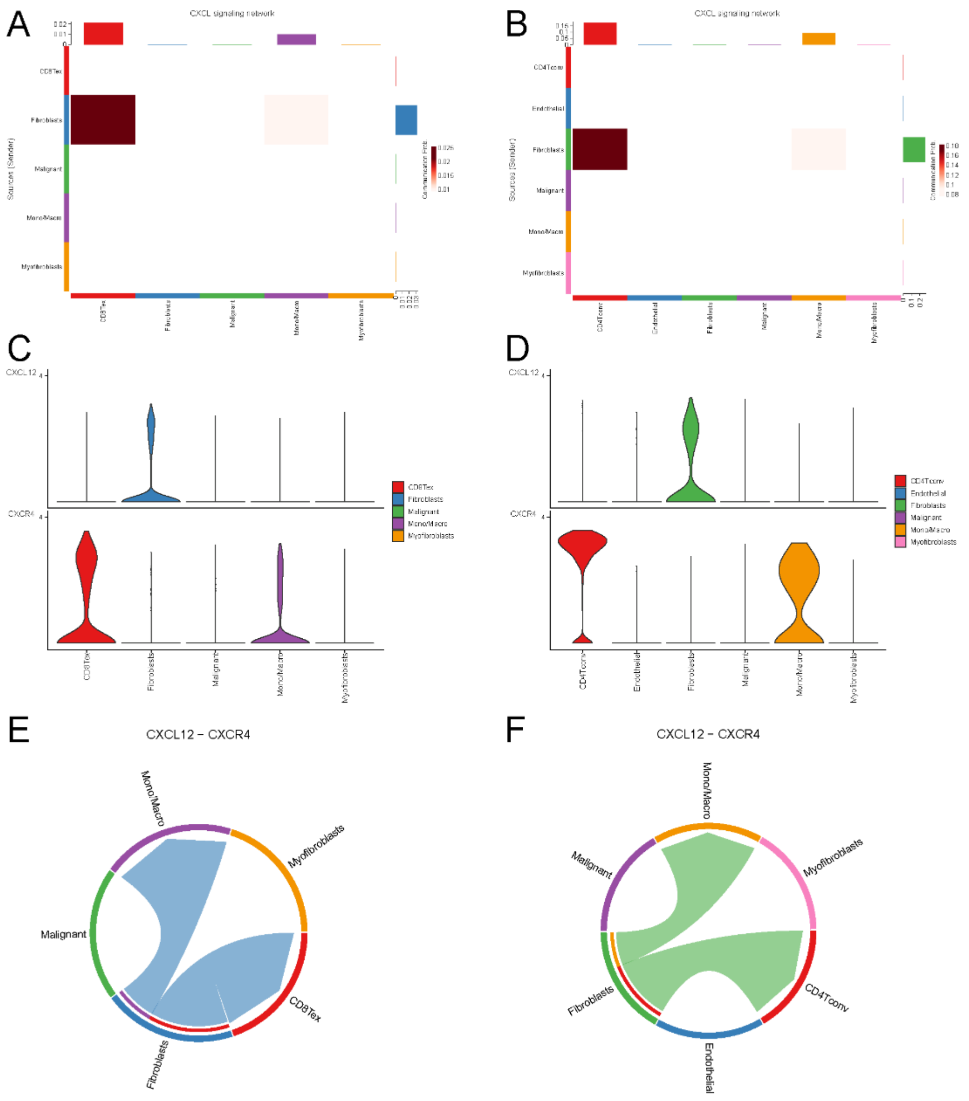
A
B
C
D
E
F
CXCL signaling network
Sources (Sender)
CD8Tex
Fibroblasts
Malignant
Mono/Macro
Myofibroblasts
CD4Tconv
Endothelial
CXCL12
CXCR4
CXCL12 - CXCR4

FigureS4

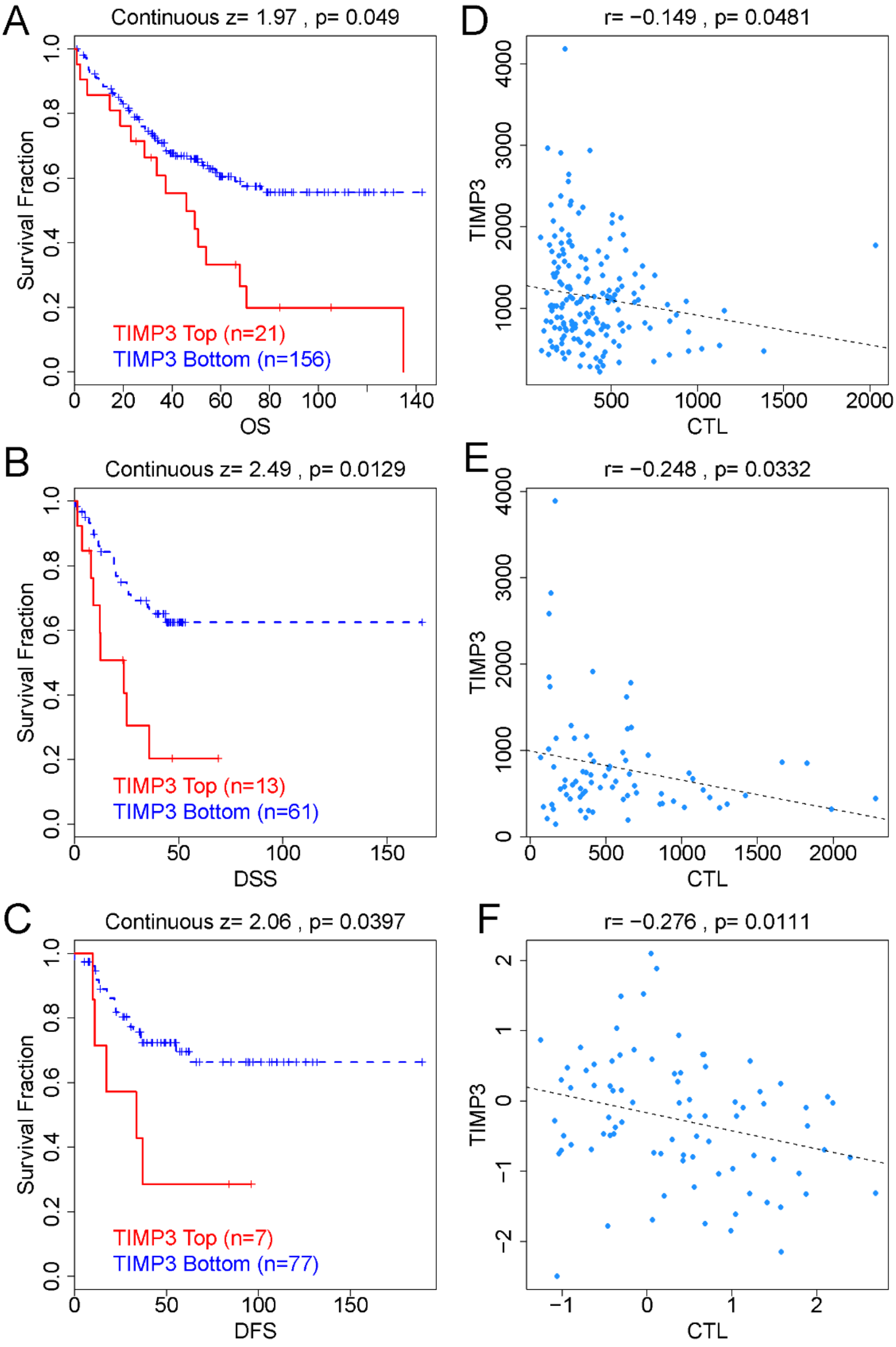
A
Continuous z= 1.97 , p= 0.049
Survival Fraction
TIMP3 Top (n=21)
TIMP3 Bottom (n=156)
OS
D
r= −0.149 , p= 0.0481
TIMP3
CTL
B
Continuous z= 2.49 , p= 0.0129
Survival Fraction
TIMP3 Top (n=13)
TIMP3 Bottom (n=61)
DSS
E
r= −0.248 , p= 0.0332
TIMP3
CTL
C
Continuous z= 2.06 , p= 0.0397
Survival Fraction
TIMP3 Top (n=7)
TIMP3 Bottom (n=77)
DFS
F
r= −0.276 , p= 0.0111
TIMP3
CTL

Figure S1 Analysis of ovarian cancer single-cell data. (A) Ovarian cancer cell cluster t-SNE (B) Cell scale diagram in ovarian cancer (C) Cell interaction network diagrams (D-F) Communication network diagrams of fibroblasts in overall, primary, and metastatic ovarian cancer cells (G) Fibroblast enrichment analysis.

Figure S2 Workflow diagram and K-M prognostic analysis. A. Selection criteria and FIB Workflow diagram of five prognostic genes, B-F. K-M prognostic analysis of 5 genes TIMP3, DUSP1, CCDC80, THY1, IFI27.

Figure S3 Communication analysis between fibroblasts and macrophages in ovarian cancer (A, B) Heatmaps were used to demonstrate that communication between fibroblasts and macrophages is dependent on the CXCL signaling pathway (C, D) Gene expression of CXCL12 in fibroblasts and expression of CXCR4 in macrophages (E, F) Intercellular cell dependent on the CXCL12-CXCR4 signaling axis inter-cellular communication of the CXCL12-CXCR4 signaling axis.

Figure S4 Patient survival analysis based on TIMP3 expression in multiple tumors (A) Prediction of OS in patients with colorectal cancer (B) Correlation analysis between TIMP3 expression and the number of CTL (cytotoxic T-lymphocytes) in colorectal cancer (C) Prediction of DSS in patients with head and neck squamous cell carcinoma (D) Correlation analysis between TIMP3 expression and the number of CTL (cytotoxic T-lymphocytes) in head and neck squamous cell carcinoma. lymphocyte) number in head and neck squamous cell carcinoma (E) Correlation analysis of predicted DFS in breast cancer patients (F) Correlation analysis of TIMP3 expression and CTL (cytotoxic T lymphocyte) number in breast cancer.
